# Evaluation of the Sepsis Flow Chip assay for the diagnosis of blood infections

**DOI:** 10.1371/journal.pone.0177627

**Published:** 2017-05-18

**Authors:** Antonio Galiana, Javier Coy, Adelina Gimeno, Noemi Marco Guzman, Francisco Rosales, Esperanza Merino, Gloria Royo, Juan Carlos Rodríguez

**Affiliations:** 1 Department of Microbiology, Hospital General Universitario de Elche, Fundación para el Fomento de la Investigación Sanitaria y Biomédica de la Comunidad Valenciana (FISABIO) Elche, Spain; 2 Department of Microbiology, Hospital General Universitario de Alicante, Instituto de Investigación Sanitaria y Biomédica de Alicante (ISABIAL - FISABIO), Alicante, Spain; 3 Department of Infectious Diseases, Hospital General Universitario de Alicante, Instituto de Investigación Sanitaria y Biomédica de Alicante (ISABIAL - FISABIO), Alicante, Spain; Cornell University, UNITED STATES

## Abstract

**Background:**

Blood infections are serious complex conditions that generally require rapid diagnosis and treatment. The big challenge is to reduce the time necessary to make a diagnosis with current clinical microbiological methods so as to improve the treatment given to patients.

**Methods:**

In this study, we assess for the first time the Sepsis Flow Chip assay, which is a novel diagnostic assay for simultaneous rapid-detection of the vast majority of bloodstream pathogens, including Gram-positive and Gram-negative bacteria and fungi, in the same assay, and for the detection of most common antibiotic resistance genes. The SFC assay is based on multiplex PCR and low density DNA arrays.

**Results:**

Positive blood cultures from 202 consecutive bacteremia patients were analyzed by SFC assay and the results were compared with the results obtained by the gold standard methodology used in clinical microbiology diagnostic laboratories (EUCAST guidelines). SFC assay overall sensitivity and specificity for bacterial identification were 93.3% and 100% respectively and sensitivity and specificity for the identification of antibiotic genetic resistance determinants were 93.6% and 100% respectively.

**Conclusions:**

This is the first evaluation of SFC assay in clinical samples. This new method appears to be very promising by combining the high number of distinct pathogens and genetic resistance determinants identified in a single assay. Further investigations should be done to evaluate the usefulness of this assay in combination with clinical multidisciplinary groups (stewardship), in order for the results to be applied appropriately to the management of patients`infectious processes.

## Introduction

Bloodstream infections are one of the most serious infectious diseases, with mortality rates of around 30–50% [1]. Factors that make bloodstream infections more difficult to manage and treat include an aging population, chronic diseases, immunosuppression, and most importantly, the increase in antibiotic resistance [2].

Although causative pathogens of bloodstream infections are consistent between studies [3], the empirical treatments used are often inappropriate [4]. This has serious clinical implications for the patient [4,5] since treatment response is directly related to the time that elapses before the appropriate antimicrobial therapy is administered. In some cases, the mortality rate can increase [6] between 6 and 10% for each hour of delay.

The conventional microbiological methods available nowadays for identifying the microorganisms causing bloodstream infections are too slow. Preliminary result based on Gram staining once the blood culture turns positive can be obtained in less than an hour but around 18 to 72 hours may be needed in order to get an accurate result including the antibiotic susceptibility pattern of the pathogen involved. In addition to the risk of administering incorrect treatment, this delay in diagnosis favors the use of broad spectrum antibiotics, implying a high healthcare cost, and the selection of antibiotic-resistant bacteria [1,3,4]. The introduction of new systems of microbiological diagnosis in clinical microbiology laboratories such as matrix-assisted mass spectrometry (MALDI-TOF) [7] or real time PCR [8] have revolutionized microbiological diagnosis producing reliable results in short periods of time. However, these new approaches have significant limitations such as the inability to detect a wide range of antibiotic resistance markers in a same test [9,10], impacting this fact in time to diagnosis.

Sepsis Flow Chip (SFC), (Master Diagnostica, Granada, Spain) is a novel microarray-based diagnostic assay for simultaneous rapid detection of microorganisms causing bloodstream infection and their most important antibiotic resistance markers directly from positive blood cultures in three hours.

The aim of this study was to evaluate for the first time the diagnostic capability of the SFC assay with a collection of microorganisms with a wide variety of genetic resistance determinants and with positive blood cultures samples.

## Material and methods

### SFC assay

SFC (Master Diagnostica, Granada, Spain) is a DNA microarray based-assay approved by the European Economic Area as a suitable device for *in vitro* diagnosis (CE IVD). SFC assay is based on a multiplex PCR amplification using biotinylated primers followed by an automatic reverse hybridization in membrane containing specific probes for detecting the most important pathogens associated with bloodstream infections and the most important genetic resistance determinants in these microorganisms (Table 1).

**Table 1:** Clinical pathogens and genetic resistant determinants detected by Sepsis Flow Chip assay.

Pathogen Identification	Genetic Resistance Determinants
**Gram-positive Bacteria**	-
*Streptococcus pneumoniae*	-
*Streptococcus agalactiae*	-
*Streptococcus spp*.	-
*Staphylococcus aureus*	*mecA*
*Staphylococcus spp*.	
*Enterococcus spp*.	*vanA/B*
*Listeria monocytogenes*	-
**Gram-negative Bacteria**	
*Stenotrophomonas maltophilia*	*blaCTX*, *blaSHV*, *blaSME*, *blaKPC*, *blaNMC/IMI*, *blaGES*, *blaIMP*, *blaGIM*, *blaVIM*, *blaSPM*, *blaSIM*, *blaNDM*, *blaOXA-23*, *blaOXA-24*, *blaOXA-48*, *blaOXA-51 and blaOXA-58*
*Serratia marcescens*	
*Escherichia coli*	
*Klebsiella pneumoniae*	
*Morganella morganii*	
*Proteus spp*.	
*Enterobacteriacea*e	
*Acinetobacter baumannii*	
*Pseudomonas aeruginosa*	
*Neisseria meningitidis*	
**Fungi**	
*Candida albicans*	-

Identification panel of SFC assay including gram-positive, gram-negative and fungi pathogens. Genetic resistance determinants identification include the main mechanisms for gram-positive pathogens and the main ESBL and carbapenemases in gram-negative pathogens.

**Fig 1:**
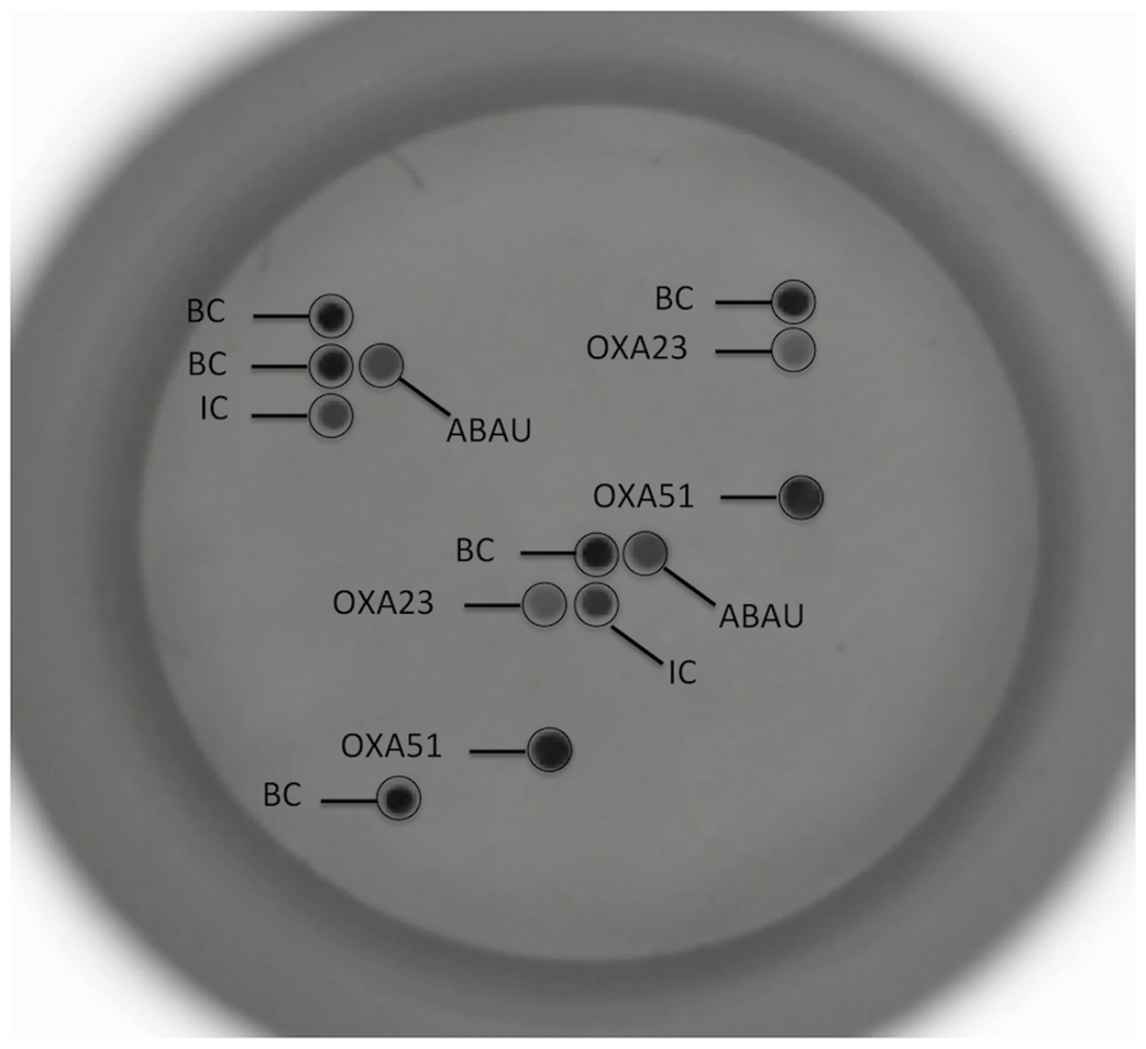
Sepsis Flow Chip device. Multi Drug Resistant *A*. *baumannii* carbapenemase producer strain analyzed by SFC assay. All target probes are by duplicate on the array and a result is considered as positive result if both signals are detected. Positive probes detected: Biotin Control (BC), Inner Control (IC), *A*. *baumannii* (ABAU), *blaOXA-23* (OXA23) and *blaOXA-51* (OXA51). The image is enlarged x10 regarding the actual size of the chip.

Positive signals are visualized via a colorimetric immunoenzymatic reaction in a chip membrane (Fig 1) by the HS24 hybridization platform. The HS24 hybridization platform has a built-in camera that captures the image of the chip and then is analized in the platform by the hybrisoft software which identifies the dot pattern that appears on the membrane. Each dot pattern is associated with a microorganism and genetic resistance determinants and the hybrisoft software provide to the user a result. The assay can detect at the species level 12 bacteria and 1 yeast, at genus level 4 bacterial genus and under the generic category “*Enterobacteriaceae”* all *Enterobacteriaceae* other than *E*. *coli*, *K*. *pneumoniae*, *M*. *morganii*, *Proteus sp*. and *S*. *marcescens*. SFC assay detects the most important genetic resistance determinants involved in resistance to methicillin and vancomycin in Gram-positive pathogens and determinants related to ß-lactam resistance mechanisms such as ESBLs and carbapenemase production in Gram-negative bacteria (Table 1). The test is performed directly from a positive blood culture using a minimum volume (10 μl).

### Pre-clinical evaluation of the SFC assay

In order to confirm the ability of the system to detect all the microorganisms and their genetic resistance determinants included in the panel, we tested a collection of 109 bacterial strains and 9 fungi strains (S1 Table) in monomicrobial or polymicrobial samples (S2 Table). The microorganisms and its genetic resitance determinants identification were previously characterized phenotypically and then confirmed genotypically. Genotypic identification were carried out by amplification of the 16S rRNA gene by PCR with 16S rRNA universal primers 27F and 1392R followed by DNA sequencing [11], 16s rDNA sequences were assigned using ribosomal database project classifier with the 16S rRNA database (https://rdp.cme.msu.edu).

Genotypic characterization of genetic resistance determinants for methicillin resistant *Staphylococcus spp*. and vancomycin resistant *Enterococcus spp*. were carried out with Xpert MRSA and Xpert VanA/vanB assay respectively (Cepheid, California, USA). Genotypic characterization of genetic resistance determinants for extended spectrum ß-lactamase (ESBL) and class A and B carbapenemase producers bacterial strains, were carried out by Real Time PCR [12,13]. Class D carbapenemase producers strains characterization were carried out by conventional PCR and DNA sequencing [14].

For pre-clinical evaluation of SFC assay, one single colony of each strain was re-suspended in distilled water at a concentration of 0.5 McFarland (1.5·108 UFC/ml) and 5 ml were inoculated in a Bactec bottle. Each sample was then manipulated as a positive blood culture following the manufacturer’s instruction prior to analysis by SFC assay. Also combinations of some samples were used for the evaluation of SFC in polymicrobial samples (S2 Table).

### Clinical evaluation of the SFC assay

Further, a total of 202 positive blood cultures obtained consecutively from unique patients with bacteremia were included in the evaluation of the SFC assay. Blood cultures from patients with bacteremia were collected in Bactec plus/F aerobic and anaerobic culture bottles (Becton Dickinson, New Jersey, USA) and incubated on BD BACTECTM 9240 (Becton Dickinson, New Jersey, USA). When the BD BACTEC system detected a positive sample, the blood culture bottle was removed from the system and analyzed in parallel by our standard microbiological methods and by SFC assay. Only one blood culture bottle per patient was analyzed by SFC regardless of whether it was an aerobic or anaerobic bottle. An aliquot of 500 μl of each blood culture sample included in the study was preserved at -20°C for future determinations in case discrepant results were obtained with the different methodologies and reproducibility assays.

According to our standard microbiological diagnostic protocols, positive blood cultures were processed for Gram staining and subcultured onto different culture media. The microorganisms were identified using MALDI-TOF (Burker Daltonics, Bremen, Germany) and this was complemented by biochemical identification and susceptibility testing by WalkAway (Beckman, San Francisco, USA) following the EUCAST guidelines [15] for analysis of antibiotic susceptibility. Confirmatory susceptibility studies were performed by E-test and by molecular methods. Methicillin resistant Staphylococci were confirmed by Xpert MRSA. Bacterial strains suspected of being carriers of ESBL or carbapenemases were confirmed by molecular methods previously described [12].

Positive blood cultures were processed in parallel by SFC assay. A volume of 10 μl of positive blood culture was diluted 1/10 with distilled water to a final volume of 100 μl and a volume of 4 μl of this dilution was used to perform the assay. Amplification reactions were carried out in a thermal cycler (Veriti Thermal Cycler, Applied Biosystems, California, USA) following manufacturer’s instructions, reverse hybridization and analysis of the results were automatically conducted with the hybriSpot HS24 platform (Master Diagnostica, Granada, Spain). The time necessary to obtain a result from a positive blood culture with SFC assay is about three hours and the platform can process up to 24 samples simultaneously.

Once the study was completed, 20 samples randomly selected from the preserved blood culture collection were reanalyzed in order to study the reproducibility of the results obtained by SFC assay.

For the evaluation of the SFC assay in positive blood cultures, sensitivity and specificity with 95% confidence intervals and concordance between conventional methods and SFC assay were calculated using SPSS v 17.0. The samples used for this study were surplus of clinical diagnosis and the data were analyzed anonymously, according to the law of biomedical research; it was not necessary to obtain informed consent. This study was approved by the local ethical committee of the Hospital General Universitario of Alicante (CEIC PI2015/39).

## Results

### Pre-clinical evaluation results

When SFC assay was tested using a collection of bacteria and fungi in monomicrobial samples, including multiresistant bacterial strains and representing all the species and genetic resistance determinants included in the test, it was seen to identify all the bacteria and *C*. *albicans* strains. Bacterial strains and other *Candida spp*. as *C*. *parapsilosis*, *C*. *tropicalis*, *C*. *krusei* and *C*. *glabrata* not included in the SFC detection panel were not detected as was expected.

SFC assay also detected 100% of bacterial strains (31/31) with genetic resistance determinants in Gram-positive bacteria and 98% (55/56) of gram-negative bacteria. The assay identified 3 *Enterococcus sp*. Strains (1 carrying *vanA* and 2 with *vanB* genes); regarding *mecA*, the assay detected correctly 28 strains (3 corresponding to *S*. *aureus* and 25 corresponding to others *Staphylococcus spp*). Similarly, SFC detected correctly 100% of ESBLs producers strains carrying *blaCTX-M* and/or *blaSHV* (28/28). Furthermore, the assay was able to detect 97% (32/33) of carbapenemase producers in strains with one or even two carbapenemases or strains with combinations of carbapenemase and ESBL. Only one carbapenemase (*blaIMP-4*) in a *K*. *pneumoniae* isolate was not detected. (S1 Table).

In polymicrobial samples the identification of all bacterial strains tested was not as good as in monomicrobial samples, the assay showed some limitations in samples with more than 3 of *Enterobacteriaceae* members. Regarding to the detection of genetic resistance determinants in polymicrobial samples, the assay showed no limitations in samples up to three microorganisms. The results of the tests performed with SFC on polymicrobial samples are shown on S2 table.

### Clinical evaluation results

Further, a total of 202 blood cultures containing 225 organisms were included in the study. One hundred and eighty eight isolates were in monomicrobial blood cultures and 37 in polymicrobial blood cultures. Of the isolates identified by MALDI-TOF and WalkAway 51.1% (115/225) were gram positive, 46.2% (104/225) were gram negative and 2.6% (6/225) were yeasts.

The gram-positive microorganisms most frequently identified by SFC assay were *Staphylococcus spp*. with 83 identifications, 13 of which were identified as *S*. *aureus*. By MALDI-TOF and WalkAway 83 isolates of *Staphylococcus spp*. were identified and distributed as follows: *S*. *epidermidis* (46/83), *S*. *hominis* (21/83) *S*. *aureus* (13/83), *S*. *capitis* (2/83) and *S*. *haemolyticus* (1/83). These results showed 100% concordance between our standard microbiological diagnostic protocols and SFC assay.

SFC assay also detected the presence of *Streptococcus spp*. in 15 blood cultures, and in 9 of these samples *S*. *pneumoniae* was identified. By MALDI-TOF and WalkAway *Streptococcus spp*. were isolated from 17 blood cultures; these isolates were identified as *S*. *pneumoniae* (9/17), *S*. *gallolyticus* (3/17), *S*. *parasanguinis* (1/17), *S*. *intermedius* (1/17), *S*. *viridans* (1/17), *S*. *salivarius* (1/17) and *S*. *oralis* (1/17). Two blood cultures harbouring *S*. *parasanguinis* and *S*. *viridans* were not detected by SFC assay. In identifying *Streptococcus spp*. the overall concordance between our standard microbiological diagnostic protocols and SFC assay was 88% (15/17) and reached 100% (9/9) for *S*. *pneumoniae*.

*Enterococcus spp*. at the genus level were also detected in 14 blood cultures by SFC assay and by MALDI-TOF and WalkAway, thus, showing a concordance between methodologies of 100% (14/14). These isolates were distributed among the species *E*. *faecalis* (13/14) and *E*. *faecium* (1/14).

Only one blood culture with *L*. *monocytogenes* was identified by SFC assay showing 100% concordance with our standard microbiological diagnostic protocols.

As for the detection of gram-negative microorganisms in positive blood cultures, the SFC assay detected at the species level the presence of *Escherichia coli*, *Klebsiella pneumoniae*, *Pseudomonas aeruginosa*, *Stenotrophomonas maltophilia*, *Serratia marcescens*, *Acinetobacter baumannii* and *Morganella morganii*. *Proteus spp*. and other *Enterobacteriaceae* were identified under the category “*Enterobacteriaceae*”. According to MALDI-TOF and WalkAway identifications, SFC assay correctly identified 100% of blood cultures containing *E*. *coli* (56/56), *K*. *pneumoniae* (15/15), *P*. *aeruginosa* (4/4) *P*. *mirabilis* (3/3), *A*. *baumannii* (3/3), *S*. *marcescens* (3/3), *S*. *maltophilia* (3/3) and *M*. *morganii* (1/1). It also correctly identified blood cultures with the presence of *K*. *oxytoca* (3/5), *Enterobacter cloacae* (4/6) and *Citrobacter freundii* (1/1) although under the generic identification of “*Enterobacteriaceae*”.

SFC assay only failed to identify *K*. *oxytoca* (2/5) and *E*. *cloacae* (2/6) in polymicrobial blood cultures, whereas there was 100% concordance between the two methodologies for these microorganisms in monomicrobial samples.

Only 4 positive blood cultures seen to be harbouring *Campylobacter jejuni*, *Bacteroides fragilis*, *Alcaligenes xylosoxidans* and *Pseudomonas putida* by MALDI-TOF and WalkAway and were negative by SFC assay since these microorganisms were not included in the assay.

Regarding the detection of yeasts in positive blood cultures, 83% of agreement was found between MALDI-TOF and SFC. All positive blood culture samples with *C*. *albicans* (5/5) identified by MALDI-TOF were detected by SFC assay and only one sample with *C*. *parapsilosis* was not detected, as expected according to the technical indications of SFC assay.

In sum, 95% of the isolates included in this study were identified by SFC assay. The details are shown in Table 2.

**Table 2:** Sensitivity and specificity of SFC assay in bacterial identification from positive blood culture samples.

Organisms isolated form positive blood cultures	No. of isolates	No. of blood culture correctly identified	Not detected	SFC result	Sensitivity	IC95%	Specificity	IC95%
**Gram-positive Bacteria**								
* Staphylococcus aureus*	13	13		*S*. *aureus*	100	71–100	100	97–100
* *Other *Staphylococcus spp*.	70	70		*Staphylococcus sp*.	100	93–100	100	97–100
* Enterococcus spp*.	14	14		*Enterococcus sp*.	100	73–100	100	97–100
* Streptococcus pneumoniae*	9	9		*S*. *pneumoniae*	100	63–100	100	98–100
* *Other *Streptococcus spp*.	8	6	2	*Streptococcus sp*.	75	35–95	100	97–100
* Listeria monocytogenes*	1	1		*L*. *monocytogenes*	75	35–95	100	97–100
**Gram-negative Bacteria**								
* Escherichia coli*	56	56		*E*. *coli*	100	92–100	100	97–100
* Klebsiella pneumoniae*	15	15		*K*. *pneumoniae*	100	74–100	100	97–100
* Klebsiella oxytoca*	5	3	2	*Enterobacteriaceae*	60	17–92	100	97–100
* Serratia marcescens*	3	3		*S*. *marcescens*	100	31–100	100	98–100
* Enterobacter cloacae*	6	4	2	*Enterobacteriaceae*	66	24–94	100	98–100
* Morganella morganii*	1	1		*M*. *morganii*	100	5–100	100	98–100
* Proteus mirabilis*	3	3		*Proteus sp*.	100	31–100	100	98–100
* Pseudomonas aeruginosa*	4	4		*P*. *aeruginosa*	100	40–100	100	98–100
* Acinetobacter baumannii*	3	3		*A*. *baumannii*	100	31–100	100	98–100
* Stenotrophomonas maltophilia*	3	3		*S*. *maltophilia*	100	31–100	100	98–100
* Citrobacter feundii*	1	1		*Enterobacteriaceae*	100	5–100	100	98–100
**Fungi**								
* Candida albicans*	5	5		*C*. *albicans*	100	46–100		97–100
* Candida parapsilosis*	1	0	1	Negative	-			-
**Not Included**								
* Campylobacter jejuni*	1	-	1	Negative				
* Bacteroides fragilis*	1	-	1	Negative				
* Achromobacter xylosoxidans*	1	-	1	Negative				
* Pseudomonas putida*	1	-	1	Negative				
**Total isolates**	225	214	11					

Sensitivity and Specificity and 95% Confidence interval of SFC assay.

When antibiotic resistance markers were analyzed for gram-positive microorganisms, SFC assay detected 52 methicillin resistant Staphylococci (*mecA* positive), as confirmed by oxacillin E-test and molecular methods as Xpert MRSA, showing 100% concordance between the two methodologies. These methicillin resistant Staphylococci were distributed between *S*. *epidermidis* (31/52), *S*. *hominis* (16/52), *S*. *capitis* (2/52), *S*. *aureus* (2/52) and *S*. *haemolyticus* (1/52). Vancomycin resistance markers such as *vanA or vanB* could not be evaluated in positive blood culture since there was a lack of positive blood cultures with vancomycin resistant *Enterococcus spp*.

In the study of antibiotic resistance in Gram-negative isolates our standard microbiological diagnosis methods as cefotaxime E-test combined with inhibitors of class A, B or D ß-lactamases identified the presence of 11 cefotaxime resistant isolates, which 9 were positive for ESBL production (Table 3) as were confirmed by molecular approach [12]. SFC assay detected 9 samples with genetic resistance determinants which could explain the 81% of cefotaxime resistant isolates. The main resistance marker associated with cefotaxime resistance identified by SFC assay was *blaCTX*, (identified in 4 blood cultures, 1 harbouring *E*. *coli* and 3 harbouring *K*. *pneumonia)*, followed by *blaSHV* identified in 2 blood cultures harbouring *E*. *coli*. Only 2 blood cultures harbouring *E*. *coli* and identified as cefotaxime resistant isolates compatible with *blaAmpC* overproduction by our standard microbiological diagnostic protocols and as were confirmed by molecular approach [13] were not detected by SFC assay since this antibiotic resistance determinant is not included in the SFC assay panel.

**Table 3:** Sensitivity and specificity of SFC assay in detection of genetic resistance determinants from positive blood culture samples.

Drug resistant organisms	No. of isolates	No. of blood culture correctly identified	Not detected	SFC result	Sensitivity (%)	IC95%	Specificity (%)	IC95%
**Gram-positive Bacteria**								
MRSA^a^	2	2		(2) *mecA*	100	18–100	100	98–100
MRCoNS^b^	50	50		(50) *mecA*	100	91–100	100	97–100
VRE^c^	0	-		-	-		-	
**Gram-negative Bacteria**								
ESBL^d^ producers	11	9	(2) *blaAmpC*	(7) *blaCTX-M*, (2) *blaSHV*	81	47–97	100	98–100
Carbapenemase producers	0	-		-	-		-	

^a^ Methicillin Resistant *S*. *aureus*.

^b^ Methicillin Resistant Coagulase Negative Staphylococci.

^c^ Vancomycin Resistant Enterococci.

^d^ Extended Spectrum β-Lactamase.

Regarding evaluation of carbapenemase detection in blood culture by SFC assay, there were only 4 blood cultures harbouring *Pseudomonas aeruginosa* carbapenem resistant isolates determined by imipenem/meroperem E-test and none were positive for carbapenemase production, as confirmed by molecular approach [13,14] (Table 3). Only 3 blood cultures harbouring *A*. *baumannii* were positive for the *blaOXA-51* resistance marker on SFC assay, although these isolates were not resistant to carbapenems as revealed by imipenem E-test.

Full concordance with previous results were found when 20 randomly selected blood cultures were reanalyzed by SFC (data not shown).

## Discussion

SFC assay was seen to be a robust, fast and easy to use tool, which can be easily implemented in clinical microbiology laboratories since it includes the most common of microorganisms normally detected in these infections [16–18] and allows the resistance mechanisms of greatest clinical importance in their treatment to be characterized. When SFC works with the hybriSpot HS24 platform, the assay is fully automated and requires a minimal sample manipulation time, which means that it may be used with positive blood cultures obtained the same workday. In this way the results are made available and may be acted upon within the first few hours of evolution of the patient’s infectious condition.

Although SFC takes a little longer to produce results than does mass spectrometry, it provides information about the presence of genetic resistance determinants in clinical material. Because antibiotic resistance is a heterogeneous process involving many factors; for example, according to our results in carbapenem resistance *P*. *aeruginosa* the absence of a carbapenemase does not imply that it is sensitive to carbapenems since other mechanisms as efflux pumps and permeability to antibiotic are involved. The lack of concordance between antibiotic susceptibility and genetic resistance determinants it is one of the main limitations of diagnosis systems based on detection of genetic resistance determinants like SFC assay.

By the other hand the detection of genetic resistance determinants directly on clinical samples in early stages of the infection is very useful when it comes to optimizing empirical treatment as quickly as possible or commencing the process of antibiotic de-escalation and will thus most likely improve the clinical management of patients [19–21]. In addition, rapid diagnosis [22,23] will make it possible to reduce the use of certain broad spectrum antibiotics and help to control the serious problem of antibiotic resistance [24] since it has been reported that hospitals are excellent compartments for the selection of resistant bacteria due to overuse and misuse of antimicrobial agents [6].

Compared to other similar systems [9] that are also currently used for diagnosis of positive blood cultures such as FilmArray BCID [21,25] or Verigene BC-GP and BC-GN [26,27], the concordance with classical methods based on culture dependent and antibiotic susceptibility testing is similar [28,29] but the main strength of SFC assay is that it provides a wider panel for identification of bacteria and genetic resistance determinants for Gram-negative organisms including the majority of carbapenemases of clinical interest described to date (*blaSME*, *blaKPC*, *blaNMC/IMI*, *blaGES*, *blaIMP*, *blaGIM*, *blaVIM*, *blaSPM*, *blaSIM*, *blaNDM*, *blaOXA-23*, *blaOXA-24*, *blaOXA-48*, *blaOXA-51*, *blaOXA-58*) in a single test [30,31]. This is of the utmost importance bearing in mind the extensive propagation in certain geographical areas of Gram negative bacteria with this resistance mechanism, in particular *Klebsiella pneumoniae* [16].

The use of this type of diagnostic method is revolutionizing the management of these patients, reducing the time they have to wait before receiving the appropriate antibiotic therapy [9,20]. However, its clinical utility is even greater if the data are managed by a multidisciplinary group (stewardship) allowing the results to be applied in an optimal manner [5,32,33]. Antimicrobial stewardship together with this type of diagnostic system also implies significant financial savings for the healthcare system since hospital and intensive care unit length of stay is reduced together with total hospital costs and even mortality [22,32].

The increase in antibiotic resistance in our hospitals [34] is an extremely serious public health problem [2,24]. Due to its complexity, it needs to be approached by multidisciplinary groups in which clinical microbiology [19] should provide fast diagnostic methods [35–37] capable of detecting the most prevalent multi-resistant microorganisms [38–40], especially those that are carbapenemase carriers, which in addition to causing infections that are difficult to treat may be associated with outbreaks in a hospital setting [41,42]. The system we evaluated will most likely prove to be a useful tool in the management of patients with bacteraemia and with the most common fungemia (*C*. *albicans*) and also help to control antibiotic resistance [5,43]. Although SFC detects fungal infections caused by *C*. *albicans*, it does not have the capacity to detect fungemia caused by other *Candida spp*. as has been shown in pre-clinical and clinical evaluation of the assay. It provides useful information for the treatment of patients just a few hours after the blood culture becomes positive [22], which is much quicker than in the case of classical microbiology, and so will help to improve the use of antibiotics. However antimicrobial susceptibility testing cannot yet be replaced by molecular systems based on identification of genetic resistance determinants, as SFC assay due to antibiotic resistance is a heterogeneous process involving many factors.

One of the main limitations of our validation study has been the lack of clinical samples with VRE and carbapenemase producer bacteria. Regarding with these major public health issue, where early report of antibiotic resistance would be very helpful for patient management by clinicians; further interventional studies with patients not only with VRE infections but also with SARM or carbapenemase producer bacteria should be done with SFC assay in combination with multidisciplinary group (stewardship) in order to see the SFC assay impact over different patients populations for example in ICU or non-ICU patients.

From our knowledge this is the first study of validation of SFC assay, SFC showed concordance with our standard microbiological diagnostic protocols of 96.2% in monomicrobial and 89.1% in polymicrobial blood cultures and an overall concordance of 92.6%. Although the results seem promising and better than to other similar diagnosis platforms, further studies with increased sample size and patient population must be done in order to evaluate other outstandings aspects that have remained in this work as detection of VRE and carbapenemase producers bacteria in blood culture.

## Supporting information

S1 Table Collection of microorganisms representing all the species and genetic resistance determinants included in the SFC assay and results obtained by SFC in pre-clinical evaluation assay. (DOCX)

S2 Table Polymicrobial samples analyzed by SFC and results obtained in pre-clinical evaluation assay. (DOCX)

## References

[1] Chalupka AN, Talmor D. The Economics of Sepsis. Critical Care Clinics. 2012. 10.1016/j.ccc.2011.09.003 22123099

[2] Hall MJ, Williams SN, DeFrances CJ, Golosinskiy A. Inpatient care for septicemia or sepsis: a challenge for patients and hospitals. NCHS Data Brief. 2011;(62). 22142805

[3] Orsini J, Mainardi C, Muzylo E, Karki N, Cohen N, Sakoulas G. Microbiological profile of organisms causing bloodstream Infection in critically Ill patients. J Clin Med Res. 2012;4(6). 10.4021/jocmr1099w PMC3513418 23226169

[4] Kollef MH. Broad-spectrum antimicrobials and the treatment of serious bacterial infections: getting it right up front. Clin Infect Dis. 2008;47. 10.1086/590061 18713047

[5] Raman G, Avendano E, Berger S, Menon V. Appropriate initial antibiotic therapy in hospitalized patients with gram-negative infections: systematic review and meta-analysis. BMC Infect Dis. 2015;15. 10.1186/s12879-015-1123-5 PMC4589179 26423743

[6] Cantón R, Horcajada JP, Oliver A, Garbajosa PR, Vila J. Inappropriate use of antibiotics in hospitals: the complex relationship between antibiotic use and antimicrobial resistance. Enferm Infecc Microbiol Clin. 2013;31. 10.1016/S0213-005X(13)70126-5 24129283

[7] Huang AM, Newton D, Kunapuli A, Gandhi TN, Washer LL, Isip J, et al Impact of rapid organism identification via matrix-assisted laser desorption/ionization time-of-flight combined with antimicrobial stewardship team intervention in adult patients with bacteremia and candidemia. Clin Infect Dis. 2013;57(9). 10.1093/cid/cit498 23899684

[8] Findlay J, Hopkins KL, Meunier D, Woodford N. Evaluation of three commercial assays for rapid detection of genes encoding clinically relevant carbapenemases in cultured bacteria. J Antimicrob Chemother. 2014;70(5). 10.1093/jac/dku571 25630646

[9] Mwaigwisya S, Assiri RAM, O’Grady J. Emerging commercial molecular tests for the diagnosis of bloodstream infection. Expert Rev Mol Diagn. 2015;15(5). 10.1586/14737159.2015.1029459 25866124

[10] García-Fernández S, Morosini MI, Marco F, Gijón D, Vergara A, Vila J, et al Evaluation of the eazyplex^®^ SuperBug CRE system for rapid detection of carbapenemases and ESBLs in clinical Enterobacteriaceae isolates recovered at two Spanish hospitals. J Antimicrob Chemother. 2014;70(4). 10.1093/jac/dku476 25428926

[11] Srinivasan R, Karaoz U, Volegova M, MacKichan J, Kato-Maeda M, Miller S, et al Use of 16S rRNA gene for identification of a broad range of clinically relevant bacterial pathogens. PLoS One. 2015;10(2). 10.1371/journal.pone.0117617 PMC4319838 25658760

[12] Roschanski N, Fischer J, Guerra B, Roesler U. Development of a Multiplex Real-Time PCR for the Rapid Detection of the Predominant Beta-Lactamase Genes CTX-M, SHV, TEM and CIT-Type AmpCs in Enterobacteriaceae. PLoS One. 2014;9(7). 10.1371/journal.pone.0100956 PMC4102473 25033234

[13] Swayne R, Ellington MJ, Curran MD, Woodford N, Aliyu SH. Utility of a novel multiplex TaqMan PCR assay for metallo-β-lactamase genes plus other TaqMan assays in detecting genes encoding serine carbapenemases and clinically significant extended-spectrum β-lactamases. Int J Antimicrob Agents. 2013;42(4). 10.1016/j.ijantimicag.2013.06.018 23988718

[14] Woodford N, Ellington MJ, Coelho JM, Turton JF, Ward ME, Brown S, Amyes SG, Livermore DM. Multiplex PCR for genes encoding prevalent OXA carbapenemases in Acinetobacter spp. Int J Antimicrob Agents. 2006;27(4). 10.1016/j.ijantimicag.2006.01.004 16564159

[15] Leclercq R, Cantón R, Brown DFJ, Giske CG, Heisig P, Macgowan AP, et al EUCAST expert rules in antimicrobial susceptibility testing. Clinical Microbiology and Infection. 2013. 10.1111/j.1469-0691.2011.03703.x 22117544

[16] Nordmann P, Dortet L, Poirel L. Carbapenem resistance in Enterobacteriaceae: Here is the storm! Trends in Molecular Medicine. 2012. 10.1016/j.molmed.2012.03.003 22480775

[17] Miriagou V, Cornaglia G, Edelstein M, Galani I, Giske CG, Gniadkowski M, et al Acquired carbapenemases in Gram-negative bacterial pathogens: Detection and surveillance issues. Clinical Microbiology and Infection. 2010. 10.1111/j.1469-0691.2009.03116.x 20085605

[18] Gould IM. MRSA bacteraemia. Int J Antimicrob Agents. 2007;30. 10.1016/j.ijantimicag.2007.06.023 17875386

[19] Avdic E, Carroll KC. The role of the microbiology laboratory in antimicrobial stewardship programs. Infectious Disease Clinics of North America. 2014. 10.1016/j.idc.2014.01.002 24857389

[20] Sothoron C, Ferreira J, Guzman N, Aldridge P, McCarter YS, Jankowski CA. A stewardship approach to optimize antimicrobial therapy through use of a rapid microarray assay on blood cultures positive for gram-negative bacteria. J Clin Microbiol. 2015;53(11). 10.1128/JCM.02161-15 PMC4609674 26292308

[21] Rödel J, Karrasch M, Edel B, Stoll S, Bohnert J, Löffler B, et al Antibiotic treatment algorithm development based on a microarray nucleic acid assay for rapid bacterial identification and resistance determination from positive blood cultures. Diagn Microbiol Infect Dis. 2015;84. 10.1016/j.diagmicrobio.2015.10.021 26712265

[22] Vardakas KZ, Anifantaki FI, Trigkidis KK, Falagas ME. Rapid molecular diagnostic tests in patients with bacteremia: evaluation of their impact on decision making and clinical outcomes. Eur J Clin Microbiol Infect Dis. 2015;34(11). 10.1007/s10096-015-2466-y 26329038

[23] Bloos F, Reinhart K. Rapid diagnosis of sepsis. Virulence [Internet]. 2014;5(1). 10.4161/viru.27393 PMC3916369 24335467

[24] Bartlett JG, Gilbert DN, Spellberg B. Seven ways to preserve the Miracle of antibiotics. Clinical Infectious Diseases. 2013. 10.1093/cid/cit070 23403172

[25] Altun O, Almuhayawi M, Ullberg M, Ozenci V. Clinical evaluation of the Filmarray blood culture identification panel in identification of bacteria and yeasts from positive blood culture bottles. J Clin Microbiol. 2013;51(12). 10.1128/JCM.01835-13 PMC3838040 24088863

[26] Wojewoda CM, Sercia L, Navas M, Tuohy M, Wilson D, Hall GS, et al Evaluation of the Verigene Gram-Positive Blood Culture Nucleic Acid Test for the Rapid Detection of Bacteria and Resistance Determinants. J Clin Microbiol. 2013;51. 10.1128/JCM.00831-13 PMC3697701 23596240

[27] Hill JT, Tran KDT, Barton KL, Labreche MJ, Sharp SE. Evaluation of the Nanosphere Verigene BC-GN assay for direct identification of gram-negative bacilli and antibiotic resistance markers from positive blood cultures and potential impact for more-rapid antibiotic interventions. J Clin Microbiol. 2014;52(10). 10.1128/JCM.01537-14 PMC4187775 25122857

[28] Bork JT, Leekha S, Heil EL, Zhao L, Badamas R, Johnson JK. Rapid testing using the verigene Gram-negative blood culture nucleic acid test in combination with antimicrobial stewardship intervention against Gram-negative bacteremia. Antimicrob Agents Chemother. 2015;59(3). 10.1128/AAC.04259-14 PMC4325795 25547353

[29] Salimnia H, Fairfax MR, Lephart PR, Schreckenberger P, DesJarlais SM, Johnson JK, et al Evaluation of the FilmArray Blood Culture Identification Panel: Results of a Multicenter Controlled Trial. J Clin Microbiol. 2016;54(3). 10.1128/JCM.01679-15 PMC4767991 26739158

[30] Kim DK, Kim HS, Pinto N, Jeon J, D’Souza R, Kim MS, et al Xpert CARBA-R assay for the detection of carbapenemase-producing organisms in intensive care unit patients of a Korean tertiary care hospital. Ann Lab Med. 2016;36(2). 10.3343/alm.2016.36.2.162 PMC4713850 26709264

[31] Nordmann P, Gniadkowski M, Giske CG, Poirel L, Woodford N, Miriagou V, et al Identification and screening of carbapenemase-producing Enterobacteriaceae. Clinical Microbiology and Infection. 2012. 10.1111/j.1469-0691.2012.03815.x 22507110

[32] Scheetz MH, Bolon MK, Postelnick M, Noskin G a, Lee T a. Cost-effectiveness analysis of an antimicrobial stewardship team on bloodstream infections: a probabilistic analysis. J Antimicrob Chemother. 2009;63(4). 10.1093/jac/dkp004 19202150

[33] Kaki R, Elligsen M, Walker S, Simor A, Palmay L, Daneman N. Impact of antimicrobial stewardship in critical care: A systematic review. Journal of Antimicrobial Chemotherapy. 2011. 10.1093/jac/dkr137 21460369

[34] Kuehn BM. Idsa: Better, faster diagnostics for infectious diseases needed to curb overtreatment, antibiotic resistance. JAMA. 2013;310(22). 10.1001/jama.2013.283828 24327022

[35] Perez KK, Olsen RJ, Musick WL, Cernoch PL, Davis JR, Peterson LE, et al Integrating rapid diagnostics and antimicrobial stewardship improves outcomes in patients with antibiotic-resistant Gram-negative bacteremia. J Infect. 2014;69(3). 10.1016/j.jinf.2014.05.005 24841135

[36] Aitken SL, Hemmige VS, Koo HL, Vuong NN, Lasco TM, Garey KW. Real-world performance of a microarray-based rapid diagnostic for Gram-positive bloodstream infections and potential utility for antimicrobial stewardship. Diagn Microbiol Infect Dis. 2015;81(1). 10.1016/j.diagmicrobio.2014.09.025 25445120

[37] Goff DA, Jankowski C, Tenover FC. Using rapid diagnostic tests to optimize antimicrobial selection in antimicrobial stewardship programs. Pharmacotherapy. 2012. 10.1002/j.1875-9114.2012.01137.x 23307517

[38] Tzouvelekis LS, Markogiannakis A, Psichogiou M, Tassios PT, Daikos GL. Carbapenemases in Klebsiella pneumoniae and Other Enterobacteriaceae: an Evolving Crisis of Global Dimensions. Clin Microbiol Rev. 2012;25(4). 10.1128/CMR.05035-11 PMC3485753 23034326

[39] Martínez-Martínez L, González-López J. Carbapenemases in Enterobacteriaceae: Types and molecular epidemiology. 2015;30. 10.1016/S0213-005X(14)70168-5 25542046

[40] Gupta N, Limbago BM, Patel JB, Kallen AJ. Carbapenem-Resistant Enterobacteriaceae: Epidemiology and Prevention. Clin Infect Dis. 2011;53(1). 10.1093/cid/cir202 21653305

[41] Hrabák J, Chudáčkova E, Papagiannitsis CC. Detection of carbapenemases in Enterobacteriaceae: a challenge for diagnostic microbiological laboratories. Clin Microbiol Infect. 2014;20(9). 10.1111/1469-0691.12678 24813781

[42] Mammina C, Bonura C, Vivoli AR, Di Bernardo F, Sodano C, Saporito MA, et al Co-colonization with carbapenem-resistant Klebsiella pneumoniae and Acinetobacter baumannii in intensive care unit patients. Scand J Infect Dis. 2013;45(8). 10.3109/00365548.2013.782614 23565771

[43] Schuts EC, Hulscher MEJL, Mouton JW, Verduin CM, Stuart JWTC, Overdiek HWPM, et al Current evidence on hospital antimicrobial stewardship objectives: A systematic review and meta-analysis. The Lancet Infectious Diseases. 2016. 10.1016/S1473-3099(16)00065-7 26947617

