# Supplementary material for: Evaluation of the Sepsis Flow Chip assay for the diagnosis of blood infections
Source: PLoS One. 2017 May 18;12(5):e0177627. doi: 10.1371/journal.pone.0177627 (PMC5436663; doi:10.1371/journal.pone.0177627)
Supplement: S1 Table — (DOCX) [file pone.0177627.s001.docx]

**Supplementary Table 1. Collection of microorganisms representing all the species and genetic resistance determinants included in the SFC assay and results obtained by SFC in pre-clinical evaluation assay, monomicrobial samples.**

| **Microorganisms tested (No of samples)** | **Genetic resistance determinants** | **SFC result** |
| --- | --- | --- |
|  |  |  |
| **Gram positive strains** |  |  |
| *E. faecium* (1) | *vanA* | *Enterococcus sp. vanA* |
| *E. faecium* (1) | *vanB* | *Enterococcus sp. vanB* |
| *E. faecalis* (1) | *vanB* | *Enterococcus sp. vanB* |
| *S. aureus* (3) | *mecA* | *S. aureus mecA* |
| *S. epidermidis* (25) | *mecA* | *Staphylococcus sp. mecA* |
| *S. pneumoniae* (5) | *-* | *S. pneumoniae* |
| *S. agalactiae* (1) | *-* | *S. agalactiae* |
| *L. monocytogenes* (1) | *-* | *L. monocytogenes* |
|  |  |  |
| **Gram negative strains** |  |  |
| *A. baumannii* (1) | *blaIMP-15 +blaOXA-51* | *A. baumannii blaIMP, blaOXA-51* |
| *A. baumannii* (1) | *blaSIM* | *A. baumannii blaSIM* |
| *A. baumannii* (1) | *blaOXA-23 + blaOXA-51* | *A. baumannii blaOXA-23, blaOXA-51* |
| *A. baumannii* (1) | *blaOXA-58* | *A. baumannii blaOXA-58* |
| *A. baumannii* (1) | *blaGES* | *A. baumannii blaGES* |
| *A. baumannii* (10) | *blaOXA-58 + blaOXA-51* | *A. baumannii blaOXA-51, blaOXA-58* |
| *A. baumannii* (2) | *blaOXA-24 + blaOXA-51* | *A. baumannii blaOXA-24, blaOXA-51* |
| *E. asburiae* (1) | *blaNMC* | *Enterobacteriaceae blaNMC* |
| *E. cloacae* (1) | *blaVIM-1 + blaCTX-M* | *Enterobacteriaceae blaVIM, blaCTX* |
| *E. coli* (1) | *blaCTX-M + blaSHV-2* | *E. coli blaCTX*, *blaSHV* |
| *E. coli* (1) | *blaVIM-2* | *E. coli blaVIM* |
| *E. coli* (1) | *blaNDM* | *E. coli blaNDM* |
| *E. coli* (1) | *blaOXA-48* | *E. coli blaOXA-48* |
| *E. coli* (2) | *blaSHV-2* | *E. coli blaSHV* |
| *E. coli* (20) | *blaCTX-M* | *E. coli blaCTX* |
| *K. oxytoca* (1) | *blaIMP-19* | *Enterobacteriaceae blaIMP* |
| *K. pneumoniae* (1) | *blaKPC-2* | *K. pneumoniae blaKPC*, *blaSHV* |
| *K. pneumoniae* (1) | *blaIMP-4 + blaSHV-2* | *K. pneumoniae blaSHV* |
| *K. pneumoniae* (1) | *blaIMP-8 + blaSHV-2* | *K. pneumoniae blaIMP, blaSHV* |
| *K. pneumoniae* (1) | *blaVIM-1 + blaSHV-4* | *K. pneumoniae blaVIM, blaSHV* |
| *K. pneumoniae* (2) | *blaOXA-48 + blaSHV-2* | *K. pneumoniae blaOXA-48, blaSHV* |
| *M. morganii* (2) | *-* | *M. morganii* |
| *N. meningitidis* (1) | *-* | *N. meningitidis* |
| *P. aeruginosa* (1) | *blaSPM* | *P. aeruginosa blaSPM* |
| *P. mirabilis* (1) | *-* | *P. mirabilis* |
| *S. maltophilia* (3) | *-* | *S. maltophilia* |
| *S. marcescens* (1) | *blaSME* | *S. marcescens blaSME* |
| **Fungal strains** |  |  |
| *C. albicans* (5) | *-* | *C. albicans* |
| *C. parapsilosis* (1) | *-* | Negative |
| *C. glabrata* (1) | *-* | Negative |
| *C. krusei* (1) | *-* | Negative |
| *C. tropicalis* (1) | *-* | Negative |
