# Supplementary material for: Evaluation of the Sepsis Flow Chip assay for the diagnosis of blood infections
Source: PLoS One. 2017 May 18;12(5):e0177627. doi: 10.1371/journal.pone.0177627 (PMC5436663; doi:10.1371/journal.pone.0177627)
Supplement: S2 Table — (DOCX) [file pone.0177627.s002.docx]

**Supplementary Table 2. Pre-clinical evaluation of SFC assay in polymicrobial samples.**

| **Microorganisms tested** | **Genetic resistance determinants** | **SFC result** |
| --- | --- | --- |
|  |  |  |
|  |  |  |
| *E. faecium + S. aureus* | *vanA + mecA* | *Enterococcus sp. vanA, S. aureus mecA* |
| *E. faecium + S. aureus* | *vanB + mecA* | *Enterococcus sp. vanB, S. aureus mecA* |
| *L. monocytogenes + S. aureus* | *mecA* | *L. monocytogenes, S. aureus mecA* |
| *A. baumannii + E. faecium* | *blaIMP-15 + blaOXA-51 + vanA* | *A. baumannii blaIMP, blaOXA-51, Enterococcus sp. vanA* |
| *A. baumannii + S. aureus* | *blaSIM + mecA* | *A. baumannii blaSIM, S. aureus mecA* |
| *K. oxytoca + S. aureus* | *blaIMP-19 + mecA* | *Enterobacteriaceae blaIMP, S. aureus mecA* |
| *K. pneumoniae + E. faecium* | *blaKPC-2 + blaSHV + vanB* | *K. pneumoniae blaSHV + blaKPC-2, Enterococcus sp. vanB* |
| *A. baumannii + E. coli* | *blaGES + blaSHV-2* | *A. baumannii blaGES, E. coli blaSHV* |
| *P. aeruginosa + E. coli* | *blaSPM + blaNDM* | *P. aeruginosa, E coli blaSPM + blaNDM* |
| *E. coli + E. cloacae + K.pneumoniae* | *blaVIM-2 + blaVIM-1 + blaCTX-M*  *blaSHV-2 + blaOXA-48* | *E. coli, K. pneumoniae, blaVIM + blaCTX-M + blaSHV + bla-OXA48* |
|  |  |  |
|  |  |  |
|  |  |  |
|  |  |  |
|  |  |  |
|  |  |  |
|  |  |  |
|  |  |  |
|  |  |  |
|  |  |  |
|  |  |  |
|  |  |  |
|  |  |  |
|  |  |  |
|  |  |  |
|  |  |  |
|  |  |  |
|  |  |  |
|  |  |  |
|  |  |  |
|  |  |  |
